# Smooth muscle cell phenotypic modulation during atherosclerosis

**DOI:** 10.1016/j.vph.2025.107570

**Published:** 2025-12-03

**Authors:** Louise Frausto, Matthew L. Scott, A. Wayne Orr, Arif Yurdagul

**Affiliations:** a Department of Molecular and Cellular Physiology, Louisiana State University Health Sciences Center at Shreveport, Shreveport, LA, USA; b Department of Pathology and Translational Pathobiology, Louisiana State University Health Sciences Center at Shreveport, Shreveport, LA, USA

**Keywords:** Vascular smooth muscle cells, Extracellular matrix, Atherosclerosis, Mechanotransduction, Plaque instability, Phenotypic modulation

## Abstract

Vascular smooth muscle cells (vSMCs) play a central role in atherosclerotic plaque development and stability through their remarkable phenotypic plasticity. In healthy vessels, contractile vSMCs maintain vascular tone and structural integrity. During atherogenesis, lipid accumulation, inflammatory cues, growth factors, and mechanical stress drive vSMC dedifferentiation, proliferation, and migration into the intima. This transition involves downregulation of contractile genes regulated by SRF-myocardin and induction of synthetic, proliferative, inflammatory, macrophage-like, or osteogenic phenotypes, mediated in part by KLF4, PDGF, TNFα, oxidized lipids, and TGFβ signaling. Mechanotransduction through integrins and ECM remodeling reinforces these phenotypic shifts, with pathological stretch, matrix stiffening, and provisional matrix deposition promoting plasticity via RhoA/ROCK, FAK, and YAP/TAZ pathways. Clonal expansion of select medial vSMCs further shapes plaque architecture, while non-coding RNAs fine-tune phenotypic modulation at the post-transcriptional level. Collectively, these processes contribute to fibrous cap thinning, impaired efferocytosis, necrotic core expansion, and vascular calcification – features of vulnerable plaques. Here, we review the molecular, mechanical, and post-transcriptional mechanisms driving vSMC phenotypic modulation in atherosclerosis, highlighting their contributions to plaque progression and instability, and discussing emerging areas that may inform future therapeutic strategies.

## 1. Introduction

Despite significant advances in diagnosis and treatment, atherosclerotic cardiovascular disease (CVD) remains the leading cause of death worldwide [1,2]. This chronic inflammatory disease can result in myocardial infarction, stroke, or sudden death. The inflammatory cascade that drives atherosclerosis begins with the accumulation of ApoB-containing lipoproteins, which promotes leukocyte recruitment and drives subsequent foam cell formation [3]. Due to lipotoxicity, the death of these foam cells accumulate within the developing necrotic core of the atherosclerotic plaque and exacerbates plaque instability [3]. Simultaneously, a fibrous cap comprised of vascular smooth muscle cells (vSMCs) forms to contain the necrotic core [4]. However, as these caps become progressively thin and fragile, they become susceptible to rupture.

**Fig. 1.**
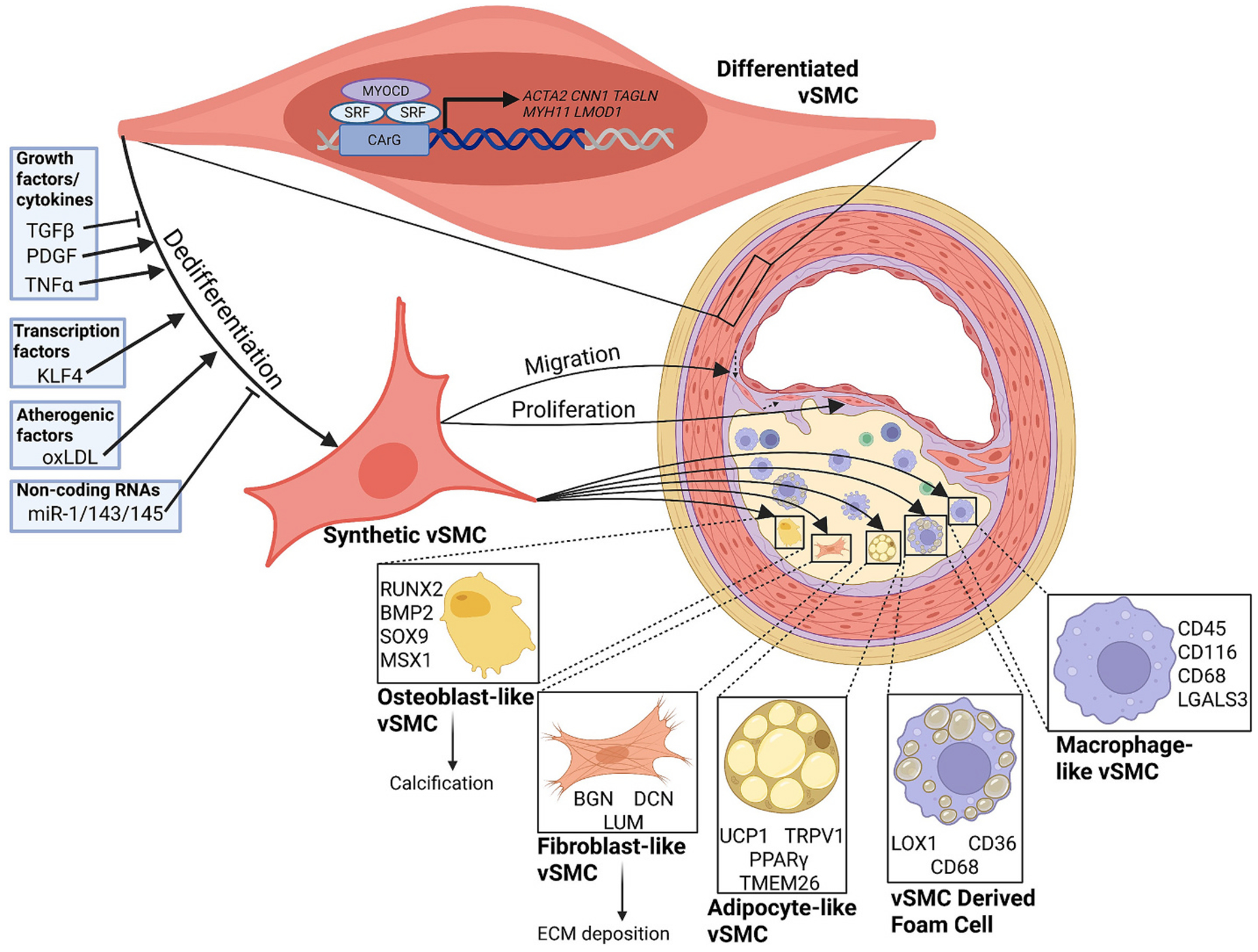
Dedifferentiation of vSMCs in Atherosclerosis. Differentiated vSMCs exhibit a contractile phenotype characterized by the expression of smooth muscle-specific contractile genes (e.g. *ACTA2*, *CNN1*, *TAGLN*, *MYH11*, and *LMOD1*) regulated by transcription factors such as SRF and its coactivator MYOCD. In response to vascular injury or atherogenic stimuli, including growth factors and cytokines (PDGF, TNFα), transcriptional regulators (KLF4), and oxidized lipids (oxLDL), vSMCs undergo differentiation into a synthetic, proliferative, and migratory state. Notably, TGFβ signaling and non-coding RNAs (miR-1/143/145) drive differentiation and inhibit dedifferentiation. Dedifferentiated vSMCs can transition into multiple cell types, adopting characteristics and gene expression patterns resembling other cells. Some adopt an osteoblast-like phenotype, characterized by the expression of *RUNX2*, *BMP2*, *SOX9*, and *MSX1*, which contributes to vascular calcification. Others adopt a fibroblast-like phenotype and deposit ECM. A subset of vSMCs differentiate into adipocyte-like cells, characterized by the expression of *UCP1*, *TRPV1*, *PPARγ*, and *TMEM26*. Additionally, some vSMCs transform into foam cells, expressing lipid-handling and macrophage-associated markers including *LOX1*, *CD36*, and *CD68*, which facilitate lipid accumulation within the plaque. Collectively, these phenotypic transitions illustrate vSMC plasticity and their central role in atherosclerotic plaque progression, remodeling and stability.

While lumen occlusion leads to angina and cardiovascular complications, most acute clinical events arise from interfacial debonding of an unstable fibrous cap overlying a necrotic core [5]. vSMC dedifferentiation, required for neointimal recruitment and fibrous cap formation, involves downregulation of contractile markers and acquisition of alternative phenotypes, some of which may subsequently contribute to plaque progression (Fig. 1) [6–11]. Unlike terminally differentiated cells, vSMCs undergo phenotypic modulation, transitioning from a contractile phenotype to one resembling fibroblasts, adipocytes, osteoblasts, or macrophages (Fig. 1) [7–9]. Under normal conditions, vSMC dedifferentiation, proliferation, and migration toward the vessel intima play a critical role in repair following vascular injury [12]. However, in atherosclerosis, dysregulation of these processes weakens the fibrous cap, promoting the formation of rupture-prone atheromas. While much is already known about the role of vSMCs in atherosclerosis, ongoing research continues to uncover new and unexpected functions, highlighting their dynamic contribution to disease progression. Gaining deeper insight into the complex role of vSMCs in atherosclerosis is critical for developing targeted therapies that can effectively halt or even reverse disease progression. Recent advances in single-cell RNA sequencing and spatial transcriptomics have further refined our understanding of vSMC plasticity by confirming the presence of heterogeneous and transitional vSMC states within human atherosclerotic plaques [13,14]. Integrating these human datasets reveals strong parallels to murine models but also important distinctions in vSMC derived phenotypes and their relative contributions to plaque stability, strengthening the transitional context. Furthermore, throughout this review, it is important to distinguish which mechanisms are supported primarily by in vitro studies versus those validated in vivo, particularly in vSMC-specific lineage-tracing models, because many widely cited pathways still lack definitive in vivo, vSMC-specific evidence. Highlighting these gaps remains essential for accurately assessing the relevance of proposed mechanisms and identifying priorities for future investigation.

Under physiological conditions, vSMCs in the tunica media actively contract and dilate to regulate blood flow through changes in vessel diameter while providing elasticity and structural support. Through crosstalk with overlying endothelial cells, vSMCs play a crucial role in distributing blood flow and regulating blood pressure in response to circulating vasoactive substances [15]. Differentiated vSMCs express high levels of contractility-related genes, including alpha-smooth muscle actin (*ACTA2*), calponin (*CNN1*), and smooth muscle protein 22-alpha (*TAGLN*), myosin heavy chain 11 (*MYH11*), and leiomodin 1 (*LMOD1*), which are essential for maintaining vascular tone (Fig. 1) [12,16]. Myocardin is a well-established coactivator of serum response factor (SRF), and together, they drive the expression of contractility-related genes in differentiated vSMCs. Recent lineage tracing studies using vSMC-specific fluorescence reporters have revealed that contractility markers decline in approximately 80 % of vSMCs during atherosclerosis. This is partially attributed to increased expression of Krüppel-like factor 4 (KLF4), which drives vSMC phenotypic switching [17–19]. Notably, contractile vSMCs are considered atheroprotective [20], and atherosclerotic plaques with a high density of vSMCs and lower infiltration by macrophages show reduced incidences of plaque rupture [10]. Here, we will review the role of vSMCs in the pathogenesis of atherosclerosis, focusing on the mechanisms behind the loss of contractility-related genes and the phenotypic fate of vSMCs in disease.

## 2. Smooth muscle hyperproliferative remodeling

VSMC proliferation is a critical initial step in atherogenesis, contributing both to neointimal expansion and to the formation of the protective fibrous cap. The various mechanisms and pathways regulating vSMC proliferation have been extensively studied for several decades and have been extensively reviewed elsewhere [21,22],. Here, we will examine the foundational regulators of vSMC proliferation and explore the novel factors that regulate the regulators. Possibly the earliest discovered mitogen observed to promote vSMC proliferation is platelet-derived growth factor, originally identified in 1973 as a platelet extract that stimulated aortic vSMC growth in culture [23]. PDGF is secreted as dimers that can consist of four different isomers (AA, AB, BB, CC, DD) [24], with the most potent dimer to act on vSMCs being the BB homodimer [25]. PDGF ligation of the PDGFRβ receptor stimulates various growth-associated pathways, including the Ras/MAPK, Pi3K/Akt, and Src-mediated signal transduction [26]. The importance of PDGF-induced vSMC growth has been demonstrated in vivo during embryonic blood vessel formation [25] and vSMC-mediated neointima formation in response to vascular injury [27–29]. The pathways and outcomes of PDGF-induced signaling in vSMCs are indeed varied and complex, and recent discoveries have added to this complexity. While PDGF is possibly the most extensively studied vSMC mitogen, other factors are also known for significant contributions to vSMC proliferation. Basic fibroblast growth factor (bFGF/FGF-2) is another vSMC mitogen that has been shown to regulate vSMC proliferation in cell culture [30,31] and neutralizing-antibody treatment reduced angioplasty-induced neointima formation *in vivo* [31,32].

In addition to growth factor mitogens, atherosclerosis-associated metabolic stress can also promote smooth muscle hyperproliferative remodleing. Atherogenesis initially begins as a fatty streak that forms in the subendothelial space and largely consists of LDL that interacts with the strata of the basement membrane where it can become oxidized (ox-LDL). vSMCs form the fibrous cap in that same subendothelial space and therefore do respond to ox-LDL by becoming more proliferative. Indeed, low to moderate doses of ox-LDL promote vSMC proliferation, while higher doses decrease cell viability [33]. Increased vSMC proliferation in response to ox-LDL treatment was shown to be though phospholipase D activation and subsequent phosphatidic acid or lysophosphatidic acid secondary messenger generation [34]. Another group demonstrated that ox-LDL stimulation of vSMCs also stimulates the sphingomyelin-ceramide-sphingosine-1-phosphate-Erk1/2 pathway in addition to the EGFR-PI3K-Akt pathway [35]. Ox-LDL treatment was also shown to suppress Tissue factor pathway inhibitor-2-mediated suppression of Cyclin D1 expression, thus promoting vSMC proliferation [36].

## 3. Smooth muscle cell dedifferentiation

VSMCs show remarkable plasticity within the atherosclerotic plaque, allowing these cells to show considerable functional variability. Much of our understanding of vSMC phenotypic modulation comes from genetic lineage-tracing systems, with *Myh11*-CreERT2 and *Tagln*-CreERT2 models firmly establishing that diverse plaque cell populations arise from medial vSMCs [17,37–41]. More recently, dual-recombinase approaches such as the Dre/rox system have enabled phenotype-specific labeling and higher-resolution fate mapping, providing clearer insight into translational vSMC states within atherosclerotic plaques [42,43]. Expression of VSMC contractility genes, *ACTA2*, *CNN1*, *TAGLN*, *MYH11*, and *LMOD1*, is governed by the master regulators myocardin, SRF, and KLF4 [44]. SRF is a master transcriptional regulator of vSMC identity, controlling the expression of contractile and other vSMC-related genes through its binding to the cis-regulatory element CC(A/T-rich)_6_GG, also termed the “CArG box [45,46]. Notably, SRF promotes vSMC differentiation due to the high abundance of CArG boxes in the promoter regions of contractility genes [47–52]. Myocardin is a key transcriptional coactivator that works alongside SRF to promote contractility gene expression [53]. KLF4 counterbalances this response by preventing the binding of SRF to CArG boxes and disrupting H4 acetylation, thus dampening contractility-related gene expression [54]. An increase in KLF4 expression can be observed in atherosclerosis, which results in the loss of vSMC-specific genes and increases proliferation, reducing fibrous cap thickness and enhancing features of plaque instability [55]. Beyond repressing contractile programs, KLF4 plays a broader role in biasing vSMC phenotypic transitions, promoting inflammatory, fibromyocyte-like, and macrophage-like states while suppressing the stabilization of fibrous-cap forming SMCs [8,17,56,57]. Recent evidence suggests that vSMC phenotypic transitions are tightly regulated by dynamic chromatin remodeling, including coordinated changes in histone acetylation and methylation that modulate the accessibility of contractile gene loci [58–60]. Dedifferentiation is associated with reductions in activating marks such as H3K27ac and increases repressive marks such as H3K27me3 at CArG-dependent promoters [61–64]. These changes are mediated by histone deacetylases (HDACs), histone methyltransferases (HMTs), and other chromatin-modifying enzymes that cooperate with KLF4 to reduce contractile gene expression [65–67].

Transforming growth factor-beta (TGFβ) is an important mediator of vSMC differentiation and expression of vSMC-specific contractility genes [68]. TGFβ activates the Smad family of intracellular signaling molecules, particularly Smad2 and Smad3, which form a complex with Smad4 [69–72]. This complex can translocate to the nucleus of vSMCs and promote the expression of vSMC-specific contractility genes [72]. Loss of TGFβ in vSMCs results in the development of unstable fibrous caps of atherosclerotic plaques through dedifferentiation involving KLF4 signaling [73]. While TGFβ promotes a contractile phenotype, mitogens and metabolic stress promote plasticity. PDGF actively promotes vSMC dedifferentiation by increasing KLF4 levels [74,75], leading to vSMC proliferation and migration [76]. Furthermore, PDGF-BB is released in response to vessel injury and activates tyrosine kinase receptors such as PDGFRβ, triggering the Ras/Raf/MEK/ERK kinase pathway [77]. This signaling cascade results in Elk-1 phosphorylation that inhibits vSMC differentiation [78,79]. Atherogenic cytokines, such as tumor necrosis factor α (TNFα), also cause vSMC dedifferentiation [20]. TNFα activates NF-κB and increases KLF4, decreasing myocardin expression and contractility-related genes, promoting vSMC proliferation, driving extracellular matrix remodeling, and enhancing proinflammatory gene expression [80–82]. Exposure to ox-LDL drives phenotypic switching of vSMCs by inducing cytokine release [83].

Upon dedifferentiation, vSMCs may pass through an intermediate synthetic or ‘pioneer’-like state, though these states have not been definitively shown to be mandatory steps in vSMC phenotypic transitions [13]. Recent single-cell RNA sequencing studies, including meta-analyses of lineage-traced murine vSMCs, have shown that this transition encompasses a spectrum of distinct phenotypic clusters rather than a single synthetic state [84,85]. In murine plaques, vSMC-derived cells segregate into contractile, fibromyocyte/ECM-producing, inflammatory, and osteochondrogenic clusters, whereas in human plaques analogous clusters exist but with greater heterogeneity, including additional ECM-remodeling, fibroblast-like, and stress response states [56,86,87]. Furthermore, vSMC dedifferentiation has been associated with increased production of proinflammatory cytokines, increased extracellular matrix (ECM) synthesis, and enhanced proliferation and migration [88]. During this dedifferentiation, vSMCs adopt a myofibroblast-like phenotype characterized by markedly increased proliferative capacity and elevated expression of ECM markers, such as fibronectin, which reinforce the connective tissue of the fibrous cap [89–91]. Notably, the loss of the contractile phenotype is associated with reduced expression of Sirtuin 1 (SIRT1), which has anti-inflammatory and anti-senescent effects in vSMCs [92].

In addition, KLF4 and ox-LDL have been linked to the transition of contractile vSMCs to macrophage-like vSMCs, a phenotype frequently associated with lipid exposure [17,20,93]. These macrophage-like cells correspond to specific inflammatory clusters identified in scRNAseq datasets, and those clusters are more prominent in mice than in human plaques, although co-expression of macrophage markers with αSMA has been observed in human lesions, supporting their translational relevance [13,17,94,95]. This transition is marked by increased expression of macrophage markers such as CD68, along with the acquisition of phagocytic function and inflammatory cytokine production [96]. Notably, co-expression of macrophage markers and αSMA has been observed in human aortic atherosclerotic plaques [97]. However, the existence and prevalence of true macrophage-like vSMCs remains a subject of debate, some studies suggesting that this phenotype may be less prominent than initially proposed. Nevertheless, to the extent that they arise, the inflammatory nature of macrophage-like vSMCs contributes to plaque weakening through impaired efferocytosis [98–100]. Additionally, continued uptake of cholesterol-rich lipoproteins by macrophage-like vSMCs can drive the progression from contractile vSMCs to foam cells [101], and the majority of foam cells found within atherosclerotic plaques are from dedifferentiated vSMCs [102,103].

Interestingly, the dedifferentiation of vSMCs into chondrocyte/osteoblast-like cells occurs during atherosclerosis [104,105], and the presence of calcification remains the strongest predictor of cardiovascular outcomes. Mechanistically, TNFα and other inflammatory mediators activate Msx2 and Wnt signaling and consequently upregulate runt-related transcription factor 2 (RUNX2) [104,106–110]. Small amounts of calcium and mineral deposits can accumulate into larger masses within the necrotic core and become calcified sheets [111]. These osteogenic-like vSMCs directly contribute to vascular calcification, driving arterial stiffness and atherosclerotic plaque instability [56,112,113]. Despite our growing understanding of the mechanisms and consequences of vSMC dedifferentiation, further elucidating the molecular mechanisms governing vSMC phenotypic modulation will be crucial for identifying therapeutic targets aimed at stabilizing atherosclerotic plaques and mitigating cardiovascular risk.

## 4. The extracellular matrix and mechanotransduction in SMC phenotype

The arterial microenvironment, including the extracellular matrix and mechanical forces generated by arterial hemodynamics, regulates multiple aspects of SMC phenotype. Vascular cells resist and respond to multiple mechanical forces, including shear stress, cyclic stretch, and hydrostatic pressure. While shear stress, the frictional force of blood flow, acts predominantly on the endothelium, SMCs resist the cyclic stretch induced by hydrostatic pressure due to the alignment of their actin cytoskeleton perpendicular to the main axis of stretch [114,115]. While physiological levels of cyclic stretch (≤10 % stretch) preserve the contractile SMC phenotype [116,117], elevated (pathological) stretch that mimics hypertension promotes SMC phenotypic plasticity, proinflammatory gene expression, and extracellular matrix remodeling [114,115]. In addition to elastin, fibrillar collagen (e.g. type I collagen, type III collagen) in the media and adventitia provide mechanical stiffness and stability to arterial tissue. Pathological arterial stiffening, commonly attributed to elastin fragmentation and accumulation of fibrillar collagens [118], can involve changes to SMC properties, such as increased cytoskeletal tension and cell-matrix interactions [119,120]. Consistent with this concept, vascular SMCs in spontaneously hypertensive rats show elevated cellular stiffness, and cytoskeletal inhibitors reduce both smooth muscle stiffness and aortic stiffness, suggesting an active role in this response [121].

Mechanical forces applied to the arterial wall (e.g. stretch, tissue stiffness) are largely transmitted to SMCs through the extracellular matrix and sensed at sites of cell-matrix adhesions, where integrins convert these forces into biochemical signals that regulate cell phenotype (a process termed mechanotransduction) [122]. Mechanotransduction-driven RhoA/ROCK signaling induces actin polymerization reducing the pool of G actin in the cell and stimulating myocardin-related transcription factors to promote the expression of SMC cytoskeletal genes (e.g. ACTA2, MYH11) [123,124]. However, pathological levels of stretch promotes SMC collagen synthesis [125,126], extracellular matrix expression, and SMC proliferation [125–127]. Cyclic stretch induces the activation of a variety of proteins known to localize to cell-matrix adhesions, such as focal adhesion kinase (FAK), paxillin, and p130Cas [128,129], all of which are associated with enhanced SMC proliferation and migration. Like cyclic stretch, arterial stiffening promotes SMC integrin signaling to induce phenotypic plasticity, extracellular matrix deposition, and proliferation. Stiff matrices promote integrin-dependent activation of the Hippo pathway transcription factor Yes-associated protein (YAP1), stimulating its nuclear translocation and interaction with TEAD family transcription factors to drive the expression of genes involved in inflammation, extracellular matrix deposition, and proliferation [130]. YAP1 shows enhanced SMC expression following arterial injury [131], and SMC YAP1 deletion results aortic aneurysm formation during development and reduced injury induced neointima formation postnataly associated with reduced SMC proliferation [132–134].

While cell-matrix interactions regulate mechanotransduction, most studies evaluating mechanotransduction in cell culture models do not adequately control for changes in matrix composition [114]. Under healthy conditions, SMCs in the media are surrounded by a thin basement membrane (composed primarily of laminin and collagen IV) [135], and interactions with this basement membrane promote SMC quiescence [136–138]. In addition, SMCs are tethered to the elastin fibers through fibrillin-1 and fibrillin-4, elastic fiber-associated proteins [139]. Mutations in fibrillin-1 associated with Marfan’s syndrome promote aortic aneurysms [140,141]. Unlike the vessel media, SMCs in remodeling vessels and in the neointima of plaques interact with fibrillar collagens, collagen VIII, and provisional matrix proteins (e.g. fibronectin, thrombospondin-1/2, osteopontin, tenascin-C) [136,142], and these interactions drive SMC phenotypic plasticity and reduced expression of contractile genes. The SMC transition to a myofibroblast-like phenotype involves increased provisional matrix expression [143], and vascular SMC adhesion to these provisional matrix proteins in culture promotes phenotypic plasticity [144–146] and proliferation [147–151], suggesting that provisional matrix deposition prevents the transition back to a contractile phenotype. Furthermore, deleting these provisional matrix proteins limits SMC fibroproliferative remodeling *in vivo* [91,152–155].

Mammals express 18 integrin α subunits and 8 β subunits, which combine to form 24 distinct integrin heterodimers with differing affinity for matrix proteins and signaling properties governing cellular phenotype [136]. Vascular SMCs express multiple collagen-binding integrins, including α1β1 and α2β1. However, α1β1 has higher affinity for collagen IV in the basement membrane [156], shows preferential expression in contractile SMCs [157], and limits SMC incorporation into atherosclerotic plaques in mice [137]. Similarly, the laminin-binding integrin α6β1 and α7β1 show highest expression in contractile SMCs [158,159], and α7β1 signaling promotes SMC quiescence and limits SMC proliferation [138,160]. Interactions between SMC α2β1 and its preferred plaque-associated collagen matrices (e.g. collagen I, collagen VIII) drives SMC proliferation and migration [161,162]. SMCs interact with provisional matrix proteins through the integrins α5β1, α8β1, and αvβ3. While α5β1 inhibition does not affect neointimal SMC levels in mouse models of atherosclerosis *in vivo* [163], transgenic overexpression of a α5/α2 chimeric integrin enhances contractile gene expression and reduces aneurysm formation in a mouse model of Marfan’s syndrome [164]. Expression of αvβ3 is enhanced during SMC phenotypic plasticity *in vitro* [165–167] and *in vivo* [168], whereas αvβ3 inhibition reduces SMC proliferation in cell culture models [149,150,169], vessel restenosis following vascular injury [151,170,171], and fibrous cap formation in atherosclerosis [172]. Unlike αvβ3, the provisional matrix-binding integrin α8β1 shows preferential expression in quiescent SMCs and appears to enhance SMC quiescence [173,174], as α8 knockout mice show enhanced restenotic vascular remodeling following injury and elevated atherosclerotic plaque formation [175]. The vascular SMC-specific expression pattern for Itga8 prompted the production of a novel Itga8-CreERT2 model, which shows minimal SMC-specific expression outside the vasculature and allows for studies in both male and female mice [176]. In medial SMCs, αvβ3 and α8β1 both interact with fibrillin to couple SMCs to the stretching elastin fibers [139,177], suggesting that alterations in integrin expression may alter stretch or stiffness-associated integrin signaling to affect SMC phenotype.

## 5. Smooth muscle clonal expansion

**Fig. 2.**
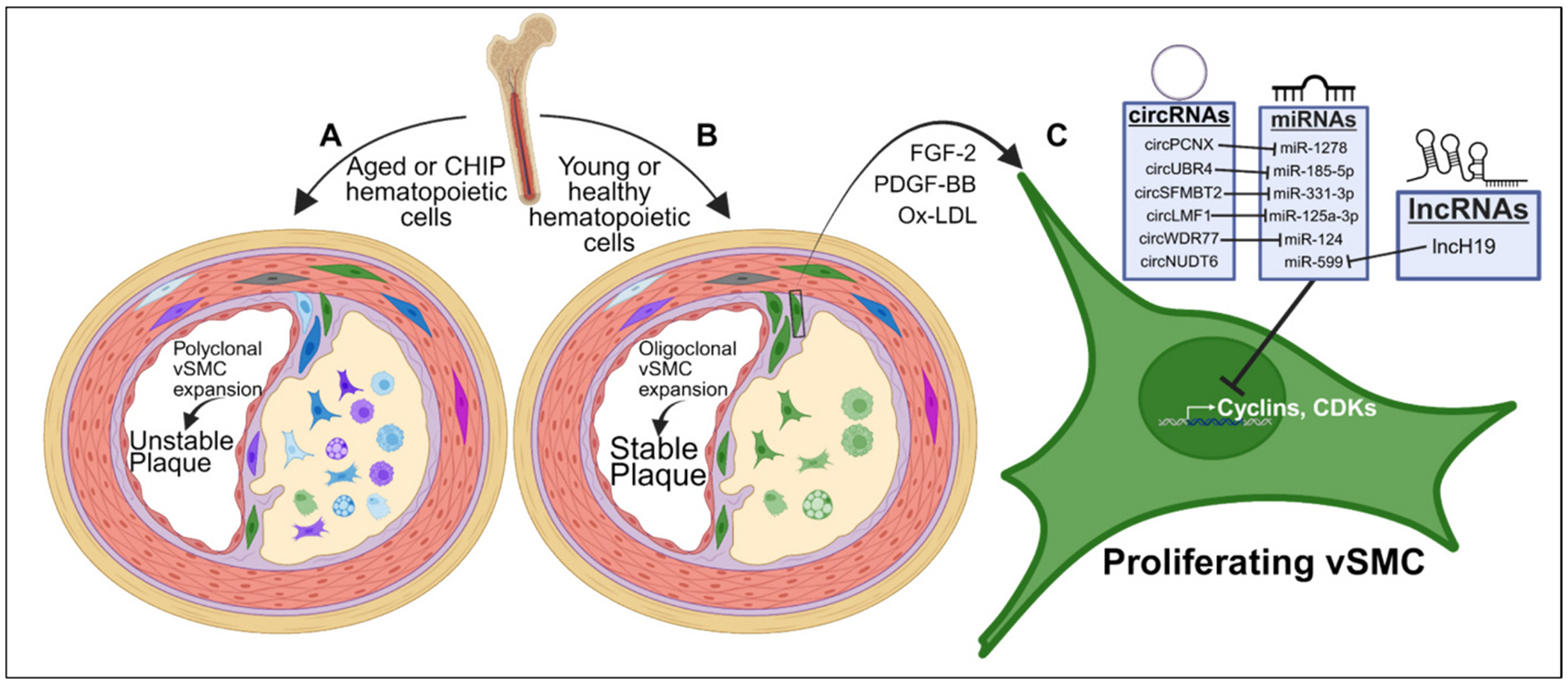
Hematopoietic cells contribute to clonal vSMC expansion in atherosclerosis. (A) Aged hematopoietic cells or those with mutations leading to clonal hematopoesis of indeterminate potential promote a polyclonal investment response from medial vSMCs whereby multiple vSMC clones integrate into the atheroma (purple, dark blue, light blue, and green cells), resulting in increased features of plaque instability (e.g. thin fibrous cap and large necrotic core). (**B**) Young or healthy hematopoietic cells promote an oligoclonal (i.e. one or two clones; green cells) vSMC investment response during atherogenesis and promoting features of plaque stabilization. (**C**) vSMC proliferation during atherogenesis can be stimulated by various factors, including PDGF-BB, FGF-2, and Ox-LDL. vSMC proliferation in response to these stimuli are multifactorial and involve the induction of various circular RNAs (circRNAs) and long non-coding RNAs (lncRNAs). These RNAs contain complimentary sequences to various anti-proliferative microRNAs (miRNAs) and function by ‘sponging’ the miRNAs, preventing them from facilitating the degradation of pro-proliferative mRNA transcripts.

An important aspect of vSMC dedifferentiation is the significant increase in proliferation that accompanies phenotypic modulation. Increased vSMC proliferation contributes to the progression of atherosclerosis by promoting neointima formation through plaque investment and expansion, although this is necessary for stabilization of atherosclerotic plaques through fibrous cap formation. vSMCs account for a significant majority of cells present within atherosclerotic plaques and are oligoclonal in their investment. The medial vSMCs present within mouse aortas are polyclonal and derived from multiple SMC progenitors [178,179]. Through the use of SMC-specific Confetti reporter tools, lesional vSMCs within mouse atheromas are confirmed to derive from one or two highly proliferative medial vSMCs that clonally expand into the various vSMC phenotypes observed during atherogenesis [38]. The monoclonal nature of vSMC investment into plaque was first discovered in humans prior to the generation of lineage-tracing mouse models initially via the analysis of X-linked gluose-6-phosphate dehydrogenase isozymes [42] and followed by the examination of methylation patterns of the X-linked human androgen receptor gene [180]. Interestingly, integrin β3 expression in bone marrow-derived cells regulates the clonal expansion of vSMCs during atherogenesis, as bone marrow transplanted from *Itgb3*^−/−^ mice into *Itgb3*^+/+^ mice exacerbates atherosclerosis and promotes the investment of multiple vSMC clones into the plaque [178]. This causal link between bone marrow-derived cells and lesional vSMC clonality was further strengthened by a study showing that bone marrow from aged mice transplanted into younger mice was sufficient to promote vSMC polyclonal expansion and drastically increase hallmarks of unstable atherosclerotic plaque (Fig. 2) [181]. The link between aged bone marrow, vSMC polyclonal expansion, and unstable atherogenesis may contribute to the recently discovered association between clonal hematopoiesis of indeterminate potential (CHIP) and de novo atherosclerosis in aged human populations [182,183]. Although a direct link between CHIP and vSMC polyclonal expansion has yet to be established in humans, mouse studies utilizing genetic deletion of the CHIP-associated gene, *Tet2*, revealed that *Tet2*^−/−^ bone marrow transplantation mimics the vSMC polyclonal expansion and worsened atherosclerosis observed with aged bone marrow transplantation [181].

## 6. Non-coding RNAs in VSMC phenotype

The large family of non-coding RNAs, such as microRNAs, circular RNAs and long non-coding (lnc) RNAs regulate multiple aspects of VSMC phenotypic transition. MicroRNAs, particularly miR-143 and miR-145, control vSMC differentiation [18,19]. These miRNAs are driven by Nk2 transcription factor related locus 5 (Nkx2–1) as well as SRF and its coactivator myocardin [19,184,185]. Oxidized LDL and cholesterol accumulation in vSMCs downregulates miR-143/145 and decreases contractility gene expression [186]. In addition to miR-143/miR-145, miR-1 represses KLF4 activity and maintains vSMCs in the contractile state [187]. Notably, miR-1 has also been identified as a marker for subclinical atherosclerosis and is downregulated in patients with asymptomatic atherosclerosis [188]. Downregulation of miR-490-3p in response to ox-LDL treatment, promotes the expression of Pappalysin-1 and insulin-like growth factor-2, both of which contribute to vSMC proliferation [189].

In addition to miRNA, changes in VSMC expression of circRNA and lncRNA can fine-tune phenotypic modulation, in part through their effects on modulating miRNA levels (Fig. 2). PDGF-BB treatment in vSMCs upregulates the circRNA pecanex homolog (circPCNX), whereas circPCNX silencing reduced PDGF-induced vSMC proliferation. PDGF-BB induced circPCNX upregulation increases DNMT1 expression due to circPCNX acting as a sponge for miR-1278, a miRNA that targets DNMT1 mRNA for degradation [190]. Another circRNA found to be upregulated by PDGF-BB treatment is circRNA lipase maturation factor 1 (circLMF1) which subsequently acted as a sponge for the microRNA, miR-125a-3p, a miRNA shown to target the mRNAs of VEGFA and FGF1 [191]. Examination of human atherectomy tissue revealed that another circRNA, circSFMBT2, was aberrantly expressed in neointimal tissue compared to healthy control tissue. The authors demonstrated that circSFMBT2 is upregulated with PDGF-BB treatment, where it binds and inhibits the function of miR-331-3p, resulting in VSMC proliferation and migration [192].The circRNA WDR77 promotes FGF-2 expression in vSMCs via sponging miR-124, a miRNA that targets FGF-2 mRNA [193]. In response to ox-LDL treatment, circUBR4 was shown to act as a sponge for miR-185-5p, which suppresses vSMC proliferation by targeting the fibroblast growth factor receptor substrate 2 mRNA for degradation [194]. FGF-2 is also a target of the long non-coding RNA Nudix Hydrolase 6 (NUDT6), which exhibits increased expression in advanced, unstable or ruptured human atherosclerotic plaques and is inversely correlated with FGF-2 [195]. Antisense targeting of NUDT6 restored FGF-2 expression in mouse models and improved fibrous cap formation [195]. The long non-coding RNA H19 was induced by ox-LDL treatment, where it acts as a sponge to miR-599, another miRNA shown to target Pappalysin-1 [196]. The lncRNA CDKN2B-AS1, also known as ANRIL, is a key element in the CAD risk-associated locus Chr9p21, is detectable in human atherosclerotic plaques [197], and is associated with proliferative vSMCs [198] via the upregulation of fibroblast grow factor receptor substrate 2 by sponging miR-339-5p. Cardiac mesoderm enhancer-associated noncoding RNA (CARMN) is a primarily vSMC-specific lncRNA detected in mice and humans that shares an expression pattern with vSMC contractile genes [199]. CARMN facilitates vSMC contractile gene expression through an interaction with myocardin, independent of miRNA interactions and its re-expression attenuated neointima formation in balloon-injured carotid arteries in rats [199]. CARMN depletion in vSMCs resulted in drastic transcriptomic changes associated with increased vSMC proliferation, migration, inflammation, and foam cell formation [200,201]. With mounting evidence demonstrating the importance of non-coding RNAs in atherosclerosis, therapeutic strategies targeting these molecules are increasingly being explored. Strategies include antisense oligonucleotides such as GapmeRs (lnc/circRNA-targeting; RNaseH1-mediated degradation) and antagomirs (miRNA-silencing), siRNA-mediated mRNA targeting, and miRNA mimics (for a comprehensive review of RNA-based therapeutics, see [202]). While RNA-targeting therapies are showing promise, there are cautions that must be observed, including: lack of tissue and cell type specificity, potential for significant off-target effects, potential immunogenicity, dosage considerations, and potential for toxicity.

## 7. Conclusion and future directions

Our understanding of SMC phenotypic modulation in atherosclerosis has evolved dramatically over the past decade. Once considered as simple structural elements that control blood flow through contractility, SMCs are now recognized as highly plastic and central contributors to both plaque stability and disease progression. The contractile-to-synthetic phenotypic transition, marked by loss of canonical cytoskeletal gene expression and acquisition of inflammatory, proliferative, macrophage-like, or osteogenic traits, has profound consequences for plaque composition and rupture risk. This switch is orchestrated by a complex interplay of transcriptional regulators, microRNAs, inflammatory cues, mechanical stress, and ECM remodeling. While the reprogramming of SMCs may initially serve a reparative function, persistent phenotypic plasticity under pathological conditions promotes matrix degradation, fibrous cap thinning, impaired efferocytosis, necrotic core expansion, and vascular calcification.

Emerging work also highlights the role of ECM stiffness and provisional matrix deposition in maintaining the synthetic SMC phenotype. Integrin-dependent mechanotransduction via RhoA/ROCK, YAP/TAZ, and FAK pathways reinforces inflammatory and proliferative gene expression programs. Adhesion to basement membrane components like laminin and collagen IV promotes SMC quiescence, while engagement with plaque-associated collagens and provisional matrix proteins drives phenotypic plasticity and clonal expansion. Notably, several of these pathways are already tractable in preclinical settings, such as the YAP inhibitor such as verteporfin, the PDGFRβ tyrosine kinase inhibitors imatinib and crenolanib, and the integrin αvβ3 antagonists cilengitide. The recognition that integrin specificity, particularly αvβ3, α5β1, and α8β1, modulates these transitions offers a tractable entry point for therapeutic intervention. However, fundamental questions remain. The precise molecular determinants that guide vSMC transitions through intermediate states, such as the mesenchymal stem cell–like modulated SMC intermediate, toward inflammatory, osteogenic, or foam cell-like fates remain incompletely defined. Moreover, the reversibility of these transitions, the role of epigenetic memory, and the spatial dynamics of clonal expansion within the plaque microenvironment are still unclear. To address these gaps, emerging technologies will be essential [203–205]. Mass spectrometry imaging (MSI) using metal-tagged antibodies, such as imaging mass cytometry and multiplexed ion beam imaging, offers unparalleled spatial resolution and multiplexing capability. These approaches can resolve distinct vSMC phenotypes and their metabolic states within intact atherosclerotic lesions, providing a systems-level view of phenotypic diversity and metabolic rewiring. Integrating MSI with spatial transcriptomics, lineage tracing, and single cell epigenomics will be crucial for mapping SMC trajectories in vivo.

Looking forward, therapeutic strategies aimed at re-stabilizing the fibrous cap by reprogramming pathologically modulated SMCs back toward a reparative or contractile phenotype represent a promising, underexplored avenue. By dissecting the crosstalk between matrix composition, mechanical forces, and cell-intrinsic transcriptional programs, future studies have the potential to identify new targets that reduce plaque instability and improve cardiovascular outcomes.

## Data Availability

No data was used for the research described in the article.
